# A comparison of six analytical disease mapping techniques as applied to West Nile Virus in the coterminous United States

**DOI:** 10.1186/1476-072X-4-18

**Published:** 2005-08-02

**Authors:** Daniel A Griffith

**Affiliations:** 1 Ashbel Smith Professor, School of Social Sciences, University of Texas at Dallas, Richardson, TX, USA

## Abstract

West Nile Virus has quickly become a serious problem in the United States (US). Its extremely rapid diffusion throughout the country argues for a better understanding of its geographic dimensions. Both 2003 and 2004 percentages of deaths by numbers of reported human cases, for the 48 coterminous US states, are analyzed with a range of spatial statistical models, seeking to furnish a fuller appreciation of the variety of models available to researchers interested in analytical disease mapping. Comparative results indicate that no single spatial statistical model specification furnishes a preferred description of these data, although normal approximations appear to furnish some questionable implications. Findings also suggest several possible future research topics.

## Background

**Figure 1:**
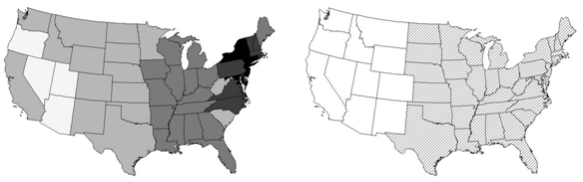
**WNV in the US**. Left: (a) diffusion of the virus: years of presence is directly proportional to darkness of the gray scale. Right: (b) 2002 presence of WNV by state, through August.

West Nile Virus (WNV [1,2]), first isolated in the West Nile District of Uganda in 1937, is a flavivirus transmitted by a mosquito vector, with a general incubation period of 2–14 days following a bite by an infected mosquito, and is closely related to the St. Louis encephalitis virus that also is found in the United States (US). WNV can infect humans, birds, mosquitoes, horses and some other mammals, with mosquitoes becoming infected after feeding on the blood of birds that carry the virus (this virus enters and circulates in a mosquito's bloodstream for a few days before it settles in the insect's salivary glands); of particular concern is that the adult WNV-carrying *Culex *species of mosquito is able to survive through winters. WNV primarily results in bird mortality, and human and equine encephalitis. In temperate latitudes, West Nile encephalitis cases occur primarily in the late summer or early fall; WNV tends to be carried by less than 1 out of every 100 mosquitoes residing in geographic regions in which it actively circulates. WNV, with its first detected US case on Long Island in 1999, has swiftly diffused across the continental US (Figure 1a and Table 1) as well as elsewhere in the Western Hemisphere. This virus enjoyed a surprisingly rapid rate of diffusion, spreading from the New York City area to nearby localities contagiously, as well as leaping across space in a hierarchical fashion through, for example, bird migration routes (see ). Although presently a person has a low risk of contracting WNV, many people infected with this virus – more than 16,000 have tested positive to date – tend to experience mild (e.g., flu-like symptoms such as fever, headache, body ache and skin rash) or no symptoms (i.e., never realizing that they have been exposed to WNV), with less than 1% of those infected developing serious illness (e.g., high fever, severe headache, stiff neck, disorientation, tremors, muscle weakness, paralysis and coma), and even fewer dying from the virus (roughly 650 to date). People at higher risk of developing potentially serious conditions include the elderly (age 50 and older) and those with lowered immune systems, and some categories of outdoor workers. Meanwhile, because 1999 signifies the beginning of a logistic growth curve for WNV cases in the US, during the very early years of the new millennium, public health officials mostly were concerned about whether or not this virus had diffused to their localities. Presence or absence was the geographic quantification of choice. But once WNV appears in an area, infected people begin to die, with human deaths and cases becoming a geographic quantification of choice. In response to this public health problem, by 2002 CDC was releasing numbers of cases and of deaths, for US states; today these data also are being released for US counties. (See .) Data aggregated by state that were extracted from this web site and used in the paper for analysis purposes appear in additional file 1a (Cases of and deaths attributed to WNV); the associated geographic connectivity matrix (i.e., matrix **C**) appears in additional file 1b (US state geographic connectivity matrix).

**Table 1:** Expansion of WNV across the coterminous US.

Year	# states reporting cases	# human cases	# human deaths
1999	4	62	7
2000	4	21	2
2001	10	50	5
2002	44	4,156	284
2003	45	9,862	264
2004	40	2,539	100

Because of the inherently geographic nature of WNV containment and eradication efforts, the objective of this paper is to summarize some initial spatial statistical analyses of the georeferenced virus in its US context using disease map modeling. In equilibrium, such features of WNV as percentage deaths should contain positive spatial autocorrelation (i.e., similar percentages of deaths should cluster together on a map) because: common local weather patterns tend to spatially cluster and partially govern mosquito population dynamics; bird populations tend to have distinct migratory routes and locational preferences; socio-economic/demographic attributes of the human population cluster geographically; and, by their very administrative unit jurisdictional nature, mosquito control programs tend to be geographically concentrated. One outcome of this work is a set of research hypotheses that should be analyzed as WNV becomes more strongly entrenched in the US geographic landscape.

## Results and discussion: a comparison of disease map modeling results

Griffith [3, pp. 78–79, 114–116] reports an analysis of the presence/absence of WNV by state for January through August, 2002 (see Figure 1b). A normal probability model approximation cannot be implemented here because the response variable is binary. The join count statistics indicate the presence of strong, positive spatial autocorrelation. A visual inspection of the map furnished by the Centers for Disease Control (CDC) indicates that only the 11 most western states in the coterminous US were absent of WNV. A Markov chain Monte Carlo (MCMC) estimated auto-logistic model reveals the presence of strong, positive spatial autocorrelation. The spatial filter logistic model (a generalized linear model specification) furnishes a third description of these data, one involving eigenvector E_1_. Estimation attempts of both spatial filter and proper conditional autoregressive (PCAR) specifications with Bayesian inference using Gibbs sampling (BUGS) repeatedly failed because of encountered phase transitions [4]. One interesting feature of the generalized linear spatial filter model is its suggestion that California (a leap across geographic space), New Mexico, Montana and Washington should have had WNV present. The end-of-the-year CDC map indeed reveals WNV presence in these states. A second interesting feature of this spatial filter model is that it out-performs both the pseudo-likelihood and the MCMC estimated auto-logistic models.

**Figure 2:**
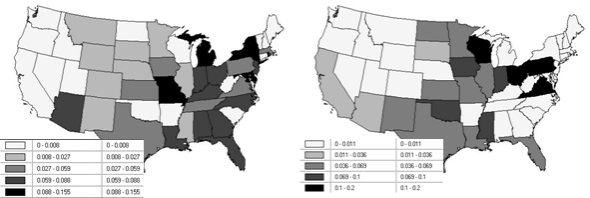
**The geographic distribution of percentages of WNV cases resulting in death**. Left (a): 2003. Right (b): 2004.

An analysis of the numbers of deaths attributable to WNV standardized by the corresponding number of reported cases (i.e., percentage data) for 2003 (overall 2.7%) and 2004 (overall 3.6%), by state (see Figure 2), reveals the presence of weak-to-negligible spatial autocorrelation (see Tables 2 and 3). An indicator variable has been included to differentiate the states without reported cases from the remaining states. Noteworthy findings here include: (1) the intercept term is approximately -3.1, regardless of model specification or year; (2) the normal probability model approximations highlight the 0-case states (3 in 2003, and 8 in 2004) as being statistically significant, whereas the remaining four specifications find them to be consistent with the other data; (3) the auto-binomial model fails to furnish a reasonable description of these 2004 data; (4) the spatial filter models indicate that both positive and negative spatial autocorrelation effects are present in these data – these countervailing geographic trends could be why the global spatial autocorrelation indices appear to be nonsignificant; and, (5) the PCAR hierarchical generalized linear model (HGLM) uncovers statistically nonsignificant spatial autocorrelation, whereas the spatial filter HGLM uncovers statistically significant spatial autocorrelation – perhaps again reflecting the mixture of positive and negative spatial autocorrelation components.

**Table 2:** Parameter estimation results for observed 2003 WNV death percentages.

	Gaussian SAR		Gaussian spatial filter		binomial spatial filter	
Statistic	estimate	se	estimate	se	estimate	se

Spatial autocorrelation	0.08	0.101	1 (spatial filter MC = 0.243)	0.144	1 (spatial filter MC = 0.359)	0.090
intercept	-3.16	0.164	-3.15	0.105	-3.11	0.078
I_0_	2.32	0.629	2.32	0.419	-18.21	25149
approximate dfs	45		39		40	
Residual P(S-W)	0.029		0.212		0.522	
Residual MC	0.0433		-0.0431 (z = -0.2)		-0.0927	
Residual GR	1.0295		0.9720		0.9782	
deviance	6.1357		4.2542		1.0198	
pseudo-R^2^	0.425		0.643		0.292	
predicted-observed regression slope	1.05		0.76		0.69	

HGLM						

	proper CAR				spatial filter	

Statistic	estimate		se		estimate	se

spatial autocorrelation	0.09		0.104		0.999 (spatial filter MC = 0.359)	0.094
intercept	- 3.25		0.187		-3.12	0.072
I_0_	-80.11		57		-78.86	60
approximate dfs	26				40	
residual P(S-W)	0.018				0.001	
residual MC	0.0119				0.0350	
residual GR	0.7141				0.7494	
deviance	0.4089				1.1660	
pseudo-R^2^	0.697				0.442	
predicted-observed regression slope	1.82				0.97	

**Table 3:** Parameter estimation results for observed 2004 WNV death percentages.

	Gaussian SAR		Gaussian spatial filter		binomial spatial filter	
Statistic	estimate	se	estimate	se	estimate	se

Spatial autocorrelation	0.01	0.101	1 (spatial filter MC = 0.030)	0.135	6.07	4.701
intercept	-3.30	0.172	-3.31	0.124	-3.48	0.182
I_0_	2.63	0.491	2.62	0.323	-20.01	41223
approximate dfs	45		39		45	
Residual P(S-W)	0.249		0.256		< 0.0001	
Residual MC	-0.0224		-0.0753 (z_MC _= -0.3)		-0.073	
Residual GR	1.1361		1.0788		1.088	
deviance	0.9001		0.4686		1.0346	
pseudo-R^2^	0.494		0.754		0.07	
predicted-observed regression slope	1.31		0.72		0.87	

HGLM						

	proper CAR				spatial filter	

Statistic	estimate	se	estimate	se		

spatial autocorrelation	-0.08		0.148		0.999 (spatial filter MC = 0.762)	0.226
intercept	-3.28		0.122		-2.99	0.122
I_0_	-81.63		62		-80.94	60
approximate dfs	45				44	
residual P(S-W)	<0.0001				0.192	
residual MC	0.0596				0.0130	
residual GR	0.9417				0.9270	
deviance	0.9715				0.4992	
pseudo-R^2^	0.328				0.411	
predicted-observed regression slope	30.60^c^				0.99	

Even after employing a variable transformation to stabilize variance, the Gaussian (i.e., normal probability) approximations continue to display marked overdispersion (i.e., excessive variability) in 2003. Residuals for these models contain only trace amounts of spatial autocorrelation, and the models themselves account for nearly half of the variability in percentage deaths from WNV. But these pseudo-R^2 ^values are inflated by the inclusion of the indicator variable denoting states with no cases. And, the simultaneous autoregressive (SAR) model predicted and observed values match well for 2003 but are underestimated by roughly 30% for 2004, whereas the spatial filter predicted values tend to be overestimated by roughly 40%, on average.

According to the 2004 data (for which pseudo- and maximum likelihood estimates are equivalent here), not only does the auto-binomial model furnish a poor description of these data, but it also is plagued by overestimation of the predicted values. Meanwhile, the spatial filter generalized linear model has residuals that contain only trace negative spatial autocorrelation, furnishes a respectable description of these data, lacks overdispersion, and provides predicted and observed values that are well matched for 2003 and overestimated for 2004.

The HGLMs have effective degrees of freedom associated with them, in order to account for parameter estimates as well as random effects estimates. Although the PCAR specification furnishes the best overall description of these data for 2003, it does so by consuming considerably more degrees of freedom. This HGLM tends to exhibit considerable underdispersion (i.e., insufficient variability), a signature of negative spatial autocorrelation. Spatial autocorrelation may well remain in the 2003 HGLM residuals, with the Moran coefficient (MC) and the Geary ratio (GR) values being inconsistent in their implications. The PCAR model also dramatically underestimates predicted probabilities. In contrast, the spatial filter HGLM produces predicted values that match well with their corresponding observed values, furnishing respectable descriptions of both data sets.

Overall, no single model specification is superior to all others. The discrepancies between specification results emphasize differences in modeling assumptions, error structure, and detailed treatment of latent spatial autocorrelation, resulting in different nuances and idiosyncrasies of the data being highlighted.

## Conclusion

Several implications can be drawn from the research summarized in this paper. Foremost is that while the initial spread of WNV across the US had a prominent geographic dimension, such a dimension has not fully materialized yet for the percentage of deaths from detected cases. In addition, the spatial filter model specification is appealing because of its simplicity, and because it is able to sense the presence of a mixture of positive and negative spatial autocorrelation components latent in the geographic distribution of WNV deaths. This model feature is not shared by the more conventional specifications. These findings suggest the following research hypothesis: spatial autocorrelation contained in a disease map has its negative components fade, and its positive components strengthen, through time. This hypothesis can be tested by evaluating the regression coefficients of spatial filter eigenvectors as annual WNV data become available. The following is a second research hypothesis suggested by Tables 2 and 3: half of the variability in percentages of WNV deaths may be accounted for with yet-to-be-determined socio-economic/demographic attribute variables. Again, this hypothesis can be tested by computing the pseudo-R^2 ^model values as annual WNV data become available. It also can be assessed by uncovering the unknown covariates. Although this is a descriptive feature of WNV here, this characterization is expected to typify expansion diffusion following an initial invasion of some territory [5]. And, the following is a third hypothesis: states without reported WNV cases are from the same statistical population as states with reported cases. This hypothesis can be tested by evaluating the zero-case indicator variable generalized linear model regression coefficient as annual WNV data become available.

Because WNV risk factors include being elderly, the size of local bird populations, and exposure to certain species of mosquitoes, geographic distributions of these groups ultimately should impact upon the geographic distribution of WNV deaths. The elderly tend to be clustered nationally, with this effect perhaps being controllable by age-standardization of case counts. The use of door and window screens, mosquito repellant, and adult, larvae and breeding site mosquito control programs tend to have socio-economic/demographic dimensions with spatial expressions. All of these factors tend to impact upon contagion diffusion, inducing positive spatial autocorrelation. Meanwhile, factors such as migratory bird routes result in leaps across geographic space (i.e., hierarchical diffusion), which initially introduce a negative spatial autocorrelation dimension into the geographic distribution of WNV deaths (i.e., a location with cases being surrounded by neighboring locations with no cases). As these diffusion paths become reinforced through cyclical repetition over time, accompanied by repeated annual waves of local contagion diffusion, a more uniform geographic distribution of WNV reservoirs should materialize, causing the negative spatial autocorrelation dimension to fade away. In the end, WNV should be characterized by positive spatial autocorrelation reflecting annual weather map patterns that promote, and the effectiveness of public health programs that attempt to minimize, the size of mosquito populations. Given the figures reported in Table 1, these patterns should contain a fair degree of variability. A similar argument pertains to horse deaths from WNV, too.

The principal covariate in the spatial statistical model specifications estimated in this paper is spatial autocorrelation. Based on the pseudo-R^2 ^values reported in Tables 2 and 3, this covariate tends to account for about 50% of the variability in the percentages of WNV deaths. This finding is a clue that socio-economic/demographic attributes – presumably associated with the use of door and window screens, mosquito repellant, and adult, larvae and breeding site mosquito control programs – should be explored in order to identify those that statistically describe this variability. Of course, part of this covariation may disappear by using age-standardized figures. Part may disappear over time as health professionals increasingly gain experience in detecting and treating WNV cases. And, part may disappear as the hierarchical diffusion component of WNV expansion disappears.

Now that WNV has appeared at one time or another in each of the coterminous US states, years in which no cases are detected in a given state have become a feasible outcome within the same statistical population. These zero-cases can naturally occur when weather patterns suppress mosquito populations, or can result from temporary effectiveness of human uses of screens and mosquito repellants, as well as governmental adult, larvae and breeding site mosquito control programs. They also can result from cyclical biological processes in the various animal populations involved in WNV transmission. When inspecting Table 1, one should not be surprised that the more severe outbreak year of 2003 is accompanied by only 3 states having zero cases, while the less severe outbreak year of 2004 is accompanied by 8 states having zero cases. Of note is that this scenario is somewhat incomplete, since the spatial diffusion of WNV was reaching geographic saturation during these two years. Nevertheless, more severe outbreak years should tend to be accompanied by a more widespread geographic distribution of the virus.

Another general finding is that the normal probability approximation model specification tends to overemphasize the statistical significance of those states with no cases. In part this result may link to the use of translation parameters that convert the odds ratio for these states to roughly 0.5. Meanwhile, one implementation data adjustment made for the other model specifications involved recoding 0s to 1s for the cases variable, which does not alter the corresponding percentage (i.e., the number of deaths remained 0). This data adjustment will be dispensed with once WNV becomes more prevalent across the US, and hence states will not be without cases, but most likely will have to be retained for initial county-level geographic resolution studies. And, because of the size of its autoregressive parameter, the auto-binomial model proves to be of less interest for model comparison purposes.

Overall general findings suggest several rules of thumb that should help guide an analyst in his/her disease map modeling efforts. Foremost, switching between model specifications should yield similar intercept values; if markedly different values are obtained, an analyst should be suspicious and ascertain why. Second, non-normal data are best described with non-normal probability models; an analyst always should be aware of nontrivial specification error. Third, a Gaussian approximation spatial filter model can be used to more quickly *explore *whether both positive and negative spatial autocorrelation components underpin a disease map; a spatial filter model specification enables a detailed understanding of latent spatial autocorrelation. And, fourth, a spatial filter can be used to more quickly *explore *spatial structuring of random effects in a Bayesian analysis involving a large n; a spatial filter model specification dramatically reduces the numerical intensity of MCMC computations.

Finally, popular individual observation diagnostic statistics may be evaluated in terms of their covariations with spatial autocorrelation by regressing them on the candidate spatial filter eigenvectors. DFBETA diagnostic statistics, one for each attribute variable, specify the standardized differences in regression estimates for assessing the effects of individual observations on the estimated regression parameters in a fitted model. And, the HI diagnostic statistic specifies the diagonal element of the hat matrix for detecting extreme points in a regressor attribute variable matrix. For the 2003 data and the reduced-form logistic regression model, eigenvectors E_1_, E_7_, E_12 _and E_26 _account for roughly 40% of the variance in the intercept DFBETA statistic, as do eigenvectors E_3_, E_7_, E_18_, E_27 _and E_29 _for the indicator variable DFBETA statistic. Eigenvectors E_1 _and E_27 _account for roughly 20% of the variance in the HI statistic. These three sets of eigenvectors overlap with those selected for the affiliated generalized linear spatial filter model only in E_1 _and E_18_. Meanwhile, for the 2004 data and the reduced-form logistic regression model, eigenvectors E_1_, E_3_, E_10_, E_15 _and E_22 _account for roughly 45% of the variance in the intercept DFBETA statistic, as do eigenvectors E_1_, E_5_, E_14_, E_19_, E_21_, E_27 _and E_29 _for the indicator variable DFBETA statistic. Eigenvectors E_4_, E_4_, E_7 _and E_24 _account for roughly 30% of the variance in the HI statistic. These three sets of eigenvectors overlap with those selected for the affiliated generalized linear spatial filter model only in E_1 _and E_15_. Future research should include scrutinizing the full battery of such diagnostics in this manner, in order to better articulate relationships between local and global information. Subsequent research also needs to address the problem of states with very few cases, whose predicted values will tend to have excessive variability, and states with very large numbers of cases, whose predicted values will tend to be significant by default.

Together these findings suggest several implications about the diffusion of WNV, whose initial spread across the coterminous US at the state level of geographic resolution now is complete. In years to come, diffusion across states will be in terms of waves of re-infection. But infill contagious diffusion still is occurring at the county and finer resolutions. Findings summarized in this paper imply that for this level of diffusion, the geographic distribution of county populations should introduce both positive and negative spatial autocorrelation components into the resulting map patterns. Furthermore, one prominent socio-economic covariate should be the difference between rural and urban locations. Others should be covariates of the willingness of local populations to accept and fund aggressive mosquito control programs, as well as individually adopting measures to prevent mosquito bites.

## Methods: spatial statistical modeling approaches

An analyst can choose from a variety of analytical spatial statistical tools to study a disease map. The first of these to be developed historically is the spatial autoregressive model, which is based upon a normal probability model, and hence requires disease map data to conform to a bell-shaped curve; often this requirement necessitates the use of a Box-Cox type of power transformation. Recent quantitative geography methodological developments have supplemented this approach with the spatial filter model specification [6]. One advantage of a spatial filter approach is that it also enables use of a generalized linear model specification [7,8], which for disease mapping purposes is based upon the binomial, Poisson, or negative binomial probability models (depending upon whether a disease map is expressed in terms of a binary, a percentage or a count variable). Recent MCMC methodology also enables the use of the binomial, Poisson, or negative binomial probability models with a spatial autoregressive specification [9-11]. In addition, MCMC has made Bayesian analysis implementable and hence more accessible, enabling researchers to estimate both conditional autoregressive [12,13] and spatial filter HGLM specifications. Because these modeling approaches involve different data assumptions, especially in terms of error, researchers interested in analytical disease mapping need a fuller appreciation of the variety of models they can employ in their analyses; six specifications are treated here, namely three conventional and their three spatial filter counterparts. Furnishing the basis for this appreciation is one of the purposes of this paper.

A sizeable part of spatial statistics is concerned with accounting for observation correlational effects arising from the geographic configuration of data. Quantitatively characterizing this configuration commonly is achieved in one of two ways: (1) establishing a geocoded coordinate for each areal unit, and then computing inter-point distances; and, (2) establishing a surface partitioning, and then constructing an n-by-n binary matrix **C**, whose cell entries are c_ij _= 1 if areal units i and j share a boundary (employing analogies with chess: if it is non-zero in length, then the linkages are referred to as the rook's case; if it is both zero and non-zero in length, then the linkages are referred to as the queen's case), and c_ij _= 0 otherwise. This queen's adjacency formulation is employed here.

### The traditional spatial autoregressive model

Spatial statistics addresses the issue of observational correlation amongst georeferenced observations, which is known as spatial autocorrelation; this type of correlation can be indexed with a MC or a GR. This autocorrelation often is positive in nature, with most phenomena exhibiting a moderate tendency for their similar values to cluster in geographic space. Occasionally, the tendency is for dissimilar values to cluster in geographic space, representing negative spatial autocorrelation.

An autoregressive model specification accounts for spatial autocorrelation by including a variable on the right-hand side of an equation that is a function of the neighboring Y values; in other words, the disease map variable, Y, appears on both sides of an equation. When coupled with regression and the normal probability model, this specification results in a covariation term characterizing spatial autocorrelation in one of two popular ways. Denoting the autoregressive parameter that captures spatial autocorrelation with ρ, a conditional autoregressive (CAR) covariance specification involves the matrix (**I **- ρ **C**), where **I **is an n-by-n identity matrix. Because matrix **C **is raised to the power 1 (i.e., only adjacent neighbors are involved in the autoregressive function), this expression is considered a first-order specification, with the autoregressive term being **CY**. Because **I **is the identity matrix, individual areal unit variance is conditionally constant.

An important matrix can be constructed from **C1**, which is the vector of number of neighbors. If the inverse of the elements of **C1 **are inserted into the diagonal of a diagonal matrix, say **D**^-1^, then **W **= **D**^-1^**C **becomes a stochastic matrix (i.e., each of its row sums equals 1). One appealing feature of this matrix is that the autoregressive term becomes **WY**, which renders averages, rather than sums, of neighboring values. Because a covariance matrix must be symmetric, a matrix **W **specification can be used with a CAR model only by making the individual areal unit variance nonconstant: (**I **- ρ **D**^-1^**C**)**D**^-1 ^= (**D**^-1 ^- ρ **D**^-1^**CD**^-1^). One appealing feature of this version is that it restricts positive values of the autoregressive parameter to the more intuitively interpretable range of 0 ≤  ≤ 1.

The SAR model (see additional files 2b: Data input and preparation for the SAR model estimation with SAS; 2b: SAR model estimation with SAS) furnishes an alternative specification that frequently is written in terms of matrix **W**. As such, its spatial covariance is a function of the matrix (**I **- ρ **CD**^-1^)(**I **- ρ**D**^-1^**C**) = (**I **- ρ **W**^T^)(**I **- ρ **W**), where T denotes matrix transpose. The resulting matrix is symmetric, is considered a second-order specification because it includes the product of two spatial structure matrices (i.e., **W**^T^**W**) – adjacent areal units as well as those having a single intervening unit are involved in the autoregressive function – and also restricts positive values of the autoregressive parameter to the more intuitively interpretable range of 0 ≤  ≤ 1.

**Figure 3:**
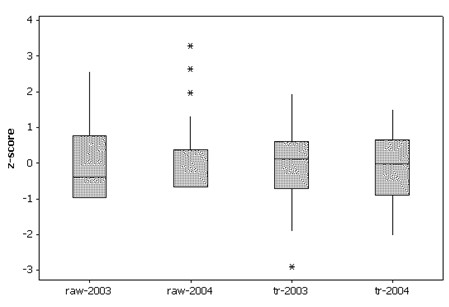
Boxplots of raw and transformed percentage deaths from diagnosed WNV cases.

For the percentage of deaths associated with diagnosed WNV cases, the log-odds ratio requires the following Box-Cox type of transformations in order to conform to a bell-shaped curve:



Non-zero translation parameters primarily are due to the presence of 0 cases, and partially due to the presence of 0 deaths for some non-zero cases. WNV deaths are a rare event, with the probability of death being highly skewed. These constants shift the locations of the empirical probabilities within the interval [0, 1] in order to have the data better conform to a normal frequency distribution. Symmetricizing effects of these transformations are portrayed in Figure 3. Zero-zero states, whose ratio becomes roughly 0.5, can be differentiated from the remaining states with an indictor variable designating them as potentially coming from a different statistical population. Because these transformations to normality should stabilize variance, arguably estimation can be done without a weighting scheme [e.g., following the variability of a binomial probability, the appropriate weighting scheme would involve division by ]; comparisons with and without the use of weights revealed no real differences in results.

### The spatial filter model

Spatial filtering involves regressing a disease map variable on a set of synthetic variates representing distinct map patterns that accounts for spatial autocorrelation; each of the three preceding spatial statistical model specifications can be replaced with a spatial filter model specification. Griffith [3] develops one form of spatial filtering whose synthetic variates are the set of n eigenvectors extracted from matrix (**I **- **11**^T^/n)**C**(**I **- **11**^T^/n), the matrix appearing in the numerator of the MC index of spatial autocorrelation, where **1 **is an n-by-1 vector of ones (see additional file 3: Minitab 14.13 code for computing spatial filter eigenvectors). This procedure is similar to executing a principal components analysis in which the covariance matrix is given by (**I **- **11**^T^/n)**C**(**I **- **11**^T^/n). But rather than using the resulting eigenvectors to construct linear combinations of attribute variables, the eigenvectors themselves (instead of principal components scores) are the desired synthetic variates, each containing n elements, one for each areal unit. The extracted eigenvector  relates to the mean response, and the remaining (n-1) extracted eigenvectors relate to distinct map patterns characterizing latent spatial autocorrelation – whose MCs are given by standardizing their corresponding eigenvalues [14] – that can materialize with matrix **C**. Furthermore, for a given geographic landscape surface partitioning, the eigenvectors represent a fixed effect in that matrix (**I **- **11**^T^/n)**C**(**I **- **11**^T^/n) does not, and hence they do not, change from one attribute variable to another.

Because this eigenfunction decomposition yields n eigenvectors, a disease map analyst needs to restrict attention to only those eigenvectors describing substantive positive/negative spatial autocorrelation (e.g., MC > 0.25 – a value that tends to relate to about 5% of the variance in Y being attributable to redundant information arising from latent spatial autocorrelation, given a particular areal unit neighborhood configuration), reducing the candidate set to a more manageable number for describing a given disease map. Supervised stepwise selection from this set of eigenvectors is a useful and effective approach to identifying the subset of eigenvectors that best describes latent spatial autocorrelation in a particular disease map. This procedure begins with only the intercept included in a regression specification. Next, at each step an eigenvector is considered for addition to the model specification. For the stepwise linear Gaussian model, commonly the eigenvector having the largest partial correlation with variable Y is selected, but only if its corresponding F-ratio achieves or surpasses a prespecified level of significance; this is the criterion used to establish statistical importance of an eigenvector. Meanwhile, in stepwise generalized linear modeling regression, the eigenvector that produces the greatest reduction in the log-likelihood function chi-square test statistic is selected, but only if it produces at least a prespecified minimum reduction; as before, this is the criterion used to establish statistical importance of an eigenvector. In each statistical procedure, at each step all eigenvectors previously entered into a spatial filter equation are reassessed, with the possibility of removal of vectors added at an earlier step. The forward/backward stepwise procedure terminates automatically when some prespecified threshold values (respectively for F-ratios and chi-square statistics) are encountered for entry and removal of all candidate eigenvectors. The ultimate inclusion criterion is determined by the MC value of the residuals, which should indicate an absence of spatial autocorrelation. Satisfying this MC condition sometimes requires supervised backward elimination of marginally selected eigenvectors because their inclusion has forced the residual MC value to decrease too far below 0. This final stopping criterion for the linear Gaussian model is relatively easy to implement because MC distributional theory is known for linear regression residuals; a corresponding stopping rule for generalized linear modeling regression is far more difficult to implement because of a lack of such distributional theory.

Spatial filters for both 2003 and 2004 maps of WNV deaths are a mixture of eigenvectors representing positive as well as negative spatial autocorrelation. The 2003 Gaussian analysis (see additional file 4: Data input, preparation, and estimation of the Gaussian spatial filter model with SAS) identifies the following as prominent eigenvectors, with the nature of their respective spatial autocorrelation denoted in parentheses:

E_1 _(+), E_2 _(+), E_6 _(-), E_10 _(+), E_15 _(-), E_21 _(-) and E_25 _(-).

Meanwhile, the 2003 generalized linear model logistic regression analysis (see additional file 5: Data input, preparation, and estimation of the logistic spatial filter model with SAS) identifies the following as prominent eigenvectors:

E_1 _(+), E_6 _(-), E_11 _(-), E_15 _(-), E_18 _(+) and E_25 _(-).

Eigenvectors E_1_, E_6_, E_15 _and E_25 _are common to these two sets. The 2004 Gaussian analysis identifies the following as prominent eigenvectors:

E_1 _(+), E_3 _(+), E_15 _(-), E_24 _(-), E_26 _(-), E_27 _(+) and E_28 _(-).

Meanwhile, the 2004 generalized linear model logistic regression analysis identifies the following as prominent eigenvectors:

E_1 _(+), E_3 _(+) and E_15 _(-).

**Figure 4:**
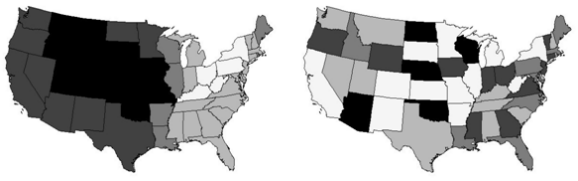
**Maps of selected eigenvectors**. Vector element values are directly proportional to darkness of the gray scale. Left (a): marked positive spatial autocorrelation (MC = 1.06). Right (b): strong negative spatial autocorrelation (MC = -0.50).

Eigenvectors E_1_, E_3 _and E_15 _are common to these two sets. Maps of eigenvectors E_1 _and E_15_, common to all four of these sets, appear in Figure 4; these represent prominent map patterns underlying the geographic distribution of WNV death percentages.

### The MCMC technique

MCMC provides a mechanism for taking *spatially dependent *samples from probability distributions in situations where the usual sampling is difficult, if not impossible. Many auto-models fall into this category, particularly because the normalizing constants for their joint or posterior probability distributions are either too difficult to calculate or analytically intractable. MCMC is used to simulate from some n-by-1 joint probability distribution **p **known only up to a constant factor, *c*. That is, **p **= *c***q**, where **q **is known but *c *is unknown and an intractable mathematical expression [see [15], pp. 428 and 431, for mathematical statements of *c *for auto-Poisson and auto-binomial models]. MCMC sampling begins with conditional (marginal) probability distributions, and parameter estimates that are obtained using pseudo-likelihood estimation (i.e., an autoregressive term is estimated with a conventional regression procedure; see additional file 6: Data input, preparation, and pseudo-likelihood estimation of the autologistic model with SAS). This involves estimating covariate coefficients (**β**) and ρ as though observations are independent. MCMC outputs a sample of values for each parameter drawn from the joint posterior probability distribution.

Gibbs sampling is a MCMC scheme for simulation from **p **where the Markov chain transition matrix (**M**) is defined by the n *conditional *probability distributions of **p**. It is a stochastic process that returns a different result with each execution, a method for generating a joint empirical distribution of several variables from a set of modeled conditional distributions for each variable when the structure of data is too complex to implement mathematical formulae or directly simulate. It is a recipe for producing a Markov chain that yields simulated data that have the correct unconditional model properties, given the conditional distributions of those variables under study [16]. The principal idea behind it is to convert a multivariate problem into a sequence of univariate problems, which then are iteratively solved to produce a Markov chain. The following Gibbs sampling algorithm description [17] is for a selected auto-model and uses the pseudo-likelihood parameter estimates of the parameters **β **and ρ:

Step 1: Initialize a map (k = 0) by taking i = 1, ..., n independent random samples {y_i,k=0_} from a chosen probability model (e.g., a binomial model).

Step 2: Obtain new values (initially k = 1) y_i,k _by sequentially moving from one location (i) to another (j) on the initial map and randomly sampling from the appropriate auto-model (e.g., the auto-binomial model) using the pseudo-likelihood parameter estimates. Site selection for this process of obtaining {y_i,k=1_} from {y_i,k=0_} can follow random permutations of location sequences. The value at each location is updated immediately after it is computed.

Step 3: Obtain new values (initially k = 2) y_i,k+1 _by sequentially moving from one location to another on the k^th ^map, again randomly sampling from the appropriate auto-model, and immediately updating the value at each location.

Step 4: Repeat step 3 for iterations k = 3, 4, ..., until convergence of the sufficient statistics of the parameters of interest occurs.

The final output then can be used to compute maximum likelihood estimates of parameters.

Once a Markov chain transition matrix is constructed, a sample of (correlated) drawings from a target distribution can be obtained. This is done by *simulating *the Markov chain a large number of times (say, 525,000), removing a "burn-in" set of iterations (say, 25,000), weeding it (select only every, say, 100^th ^result), and recording its sufficient statistics. Convergence needs to be monitored, and hence the sufficient statistics need to be recorded. This recording should be done after the completion of each iteration. A suitable burn-in period is needed in order to generate **M**, the limiting Markov chain transition probability matrix, and hence before collecting statistics, and because samples are serially correlated, the chain needs to be weeded.

One difficulty with estimation of an auto-binomial model, which is supported by MCMC techniques, is that the relationship between its intercept and autoregressive parameter is established by the global percentage of deaths for a disease map. The ideal situation occurs when ρ = -α/2 [[18], p. 3]; this point is illustrated here in Table 3. Because this parameter estimate is inconsistent with the other model specifications, this model is not treated in great detail here. Of note is that the WinBUGS software package cannot be used to estimate this model because the spatial lag variable must be recomputed at each MCMC step.

**Figure 5:**
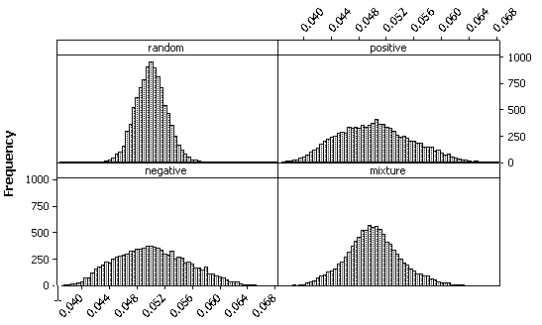
**Frequency distributions for simulated binomial probabilities (N = 10,000, p = 0.05, 100-by-100 lattice)**. Top left (a): random simulated data. Top right (b): simulated data with marked positive spatial autocorrelation embedded (MC = 1.01). Bottom left (c): simulated data with marked negative spatial autocorrelation embedded (MC = -1.01). Bottom right (d): simulated data with an equal mixture of marked positive and marked negative spatial autocorrelation embedded.

The impact of spatial autocorrelation on the frequency distribution of a binomial random variable is illustrated in Figure 5. For a 100-by-100 lattice, pseudo-random binomial counts were generated for N = 1,000 and p = 0.05; each count then was divided by 10,000. As conventional statistical theory states, the distribution of these values is approximately normally distributed with mean 0.05 and variance (0.05)(1-0.05)/100. The principal impact of spatial autocorrelation is to reduce the more central frequencies and increase the tail frequencies. As spatial statistical theory states, the mean remains 0.05, but the variance increases (here by a factor of 1.55), regardless of whether only positive or only negative spatial autocorrelation is contained in the probabilities. The effect of mixing an equal amount of positive and negative spatial autocorrelation is to have some of the autocorrelation effects cancel out; now the variance is inflated by a factor of 1.35, with the equal mixture better preserving the original kurtosis.

### The spatial Bayesian HGLM

Meanwhile, Bayesian random-effects HGLM specifications also can be used to deal with non-normal data. One appealing feature of this approach is that spatial autocorrelation in a non-normal georeferenced random variable can be captured without having to derive an explicit multivariate generalization of its distributional form. WinBUGS [12] and GeoBUGS [13] support implementation of this model for disease mapping purposes. When analyzing maps of disease, the random effects can be spatially structured and/or unstructured. The CAR model is one way spatial structuring is included; the spatial filter is another way. If the autoregressive parameter ρ is estimated, the specification is called a PCAR model. If the autoregressive parameter value is set equal to 1, then a second unstructured random effect term is included, and the specification is called an improper CAR (ICAR) model. Parameters are estimated with MCMC techniques.

Because Bayesian statistical analysis is involved, prior distributions need to be posited for each varying quantity: the response variable, each variable coefficient, the spatial autoregressive parameter, the error variance, and the random error term. The response variable prior distribution includes the model statement. The random error term may be posited as a PCAR or an ICAR specification. If either a ICAR or a spatial filter term is included, then a prior distribution for an unstructured random effect must be included. For a disease map, the response variable prior distribution frequently will be Poisson (for counts) or binomial (for presence/absence or percentages). The accompanying CAR/PCAR frequently involves an auto-normal specification. The spatial autoregressive parameter prior distribution usually is uniform. The normal distribution furnishes a feasible prior distribution for covariate coefficients, including the spatial filter. And, the error variance prior distribution often is the gamma distribution.

The following binomial HGLM involving the PCAR model was estimated using the US WNV data and GeoBUGS (see additional file 7: 2003 data input and estimation of the PCAR logistic spatial filter model with GeoBUGS):



where α is the intercept, I_0 _is the binary 0–1 indicator variable for 0-case states, β_1 _denotes its regression coefficient, and ν_i _denotes unobserved US state-specific random effects. The prior distributions attached to this log-mean response equation are:

D_i _~ binomial(p_i_, C_i_),

α ~ normal(0, 0.0001),

β_1 _~ normal(0, 0.0001),

ν_i _~ auto-normal , with a conditional autoregressive model specification corresponding to a proper multivariate Gaussian distribution with a full-rank covariance matrix (**I **- ρ **C**),

 ~ gamma(0.5, 0.0005), and

ρ ~ uniform(1/λ_48_, 1/λ_1_), where λ_48 _and λ_1 _respectively are the smallest and largest eigenvalues of matrix **C**,

where ~ denotes "distributed as," D_i _and C_i _respectively denote the number of deaths and the number of cases in state i, and σ_ε _is the standard deviation of the random effects term.

The spatial filter version (see additional file 8: 2003 data input and estimation of the logistic spatial filter model with WinBUGS) removes the CAR specification, and adds a spatial filter term together with its regression coefficient β_2 _~ normal(0, 0.0001); the random effects now are independent normal(0, ).

## Supplementary Material

Additional File 1 **Cases of and deaths attributed to WNV; US state geographic connectivity matrix. **Tabulated state-by-attribute data (a), and tabulated state-by-state binary geographic connectivity matrix data (b). Click here for file

Additional File 2 **Data input and preparation for the SAR model estimation with SAS; SAR model estimation with SAS. **SAS computer code, in which the input data file paths and file names may need to be changed (a), for estimating a simultaneous spatial autoregressive model (b). Click here for file

Additional File 3 **Minitab 14.13 code for computing spatial filter eigenvectors. **Minitab computer code, in which the input data file paths and file names may need to be changed; the number of areal units is stored in K1, the geographic connectivity matrix is stored in M1, and the spatial filter eigenvectors are stored in M2. Click here for file

Additional File 4 **Data input, preparation, and estimation of the Gaussian spatial filter model with SAS. **SAS computer code, in which the input data file paths and file names may need to be changed, for estimating a linear regression spatial filter model. Click here for file

Additional File 5 **Data input, preparation, and estimation of the logistic spatial filter model with SAS. **SAS computer code, in which the input data file paths and file names may need to be changed, for estimating a generalized linear (logistic) regression spatial filter model. Click here for file

Additional File 6 **Data input, preparation, and pseudo-likelihood estimation of the auto-logistic model with SAS. **SAS computer code, in which the input data file paths and file names may need to be changed, for estimating a generalized linear auto-logistic regression model. Click here for file

Additional File 7 2003 data input and estimation of the PCAR logistic spatial filter model with GeoBUGS. Click here for file

Additional File 8 2003 data input and estimation of the logistic spatial filter model with WinBUGS. Click here for file
