# Supplementary material for: A comparison of six analytical disease mapping techniques as applied to West Nile Virus in the coterminous United States
Source: Int J Health Geogr. 2005 Aug 2;4:18. doi: 10.1186/1476-072X-4-18 (PMC1215506; doi:10.1186/1476-072X-4-18)
Supplement: Additional File 1 — Cases of and deaths attributed to WNV; US state geographic connectivity matrix. Tabulated state-by-attribute data (a), and tabulated state-by-state binary geographic connectivity matrix data (b). [file 1476-072X-4-18-S1.pdf]

1a: Cases of and deaths attributed to WNV.

US coterminous state	state zip code abbreviation	2003		2004	
		cases	deaths	cases	deaths
Alabama	AL	37	3	15	0
Arizona	AZ	13	1	391	14
Arkansas	AR	25	0	22	0
California	CA	3	0	771	23
Colorado	CO	2,947	63	276	3
Connecticut	CT	17	0	1	0
Delaware	DE	17	2	0	0
Florida	FL	94	6	41	2
Georgia	GA	50	4	19	0
Idaho	ID	1	0	3	0
Illinois	IL	54	1	57	3
Indiana	IN	47	4	12	1
Iowa	IA	147	6	22	2
Kansas	KS	91	4	43	2
Kentucky	KY	14	1	7	0
Louisiana	LA	124	8	102	7
Maine	ME	0	0	0	0
Maryland	MD	73	8	16	0
Massachusetts	MA	17	1	0	0
Michigan	MI	19	2	16	0
Minnesota	MN	148	4	34	2
Mississippi	MS	87	1	51	4
Missouri	MO	64	8	37	2
Montana	MT	222	4	6	0
Nebraska	NE	1,942	29	49	0
Nevada	NV	2	0	44	0
New-Hampshire	NH	3	0	0	0
New-Jersey	NJ	34	3	1	0
New-Mexico	NM	209	4	88	4
New-York	NY	71	11	10	0
North-Carolina	NC	24	2	3	0
North-Dakota	ND	617	5	20	1
Ohio	OH	108	8	12	2
Oklahoma	OK	79	0	20	2
Oregon	OR	0	0	3	0
Pennsylvania	PA	237	8	15	2
Rhode-Island	RI	7	1	0	0
South-Carolina	SC	6	0	1	0
South-Dakota	SD	1,039	14	51	1
Tennessee	TN	26	1	14	0
Texas	TX	720	37	158	8
Utah	UT	1	0	11	0
Vermont	VT	3	0	0	0
Virginia	VA	26	1	5	1
Washington	WA	0	0	0	0
West-Virginia	WV	2	0	0	0
Wisconsin	WI	17	0	12	2
Wyoming	WY	375	9	10	0
TOTAL		9,859	264	2,469	88

1b: US state geographic connectivity matrix.

State	binary 0-1 indicator of queen’s case adjacency
AL	0 0 0 0 0 0 0 1 1 0 0 0 0 0 0 0 0 0 0 0 0 1 0 0 0 0 0 0 0 0 0 0 0 0 0 0 0 0 0 1 0 0 0 0 0 0 0 0
AZ	0 0 0 1 0 0 0 0 0 0 0 0 0 0 0 0 0 0 0 0 0 0 0 0 0 1 0 0 1 0 0 0 0 0 0 0 0 0 0 0 0 1 0 0 0 0 0 0
AR	0 0 0 0 0 0 0 0 0 0 0 0 0 0 0 1 0 0 0 0 0 1 1 0 0 0 0 0 0 0 0 0 0 1 0 0 0 0 0 1 1 0 0 0 0 0 0 0
CA	0 1 0 0 0 0 0 0 0 0 0 0 0 0 0 0 0 0 0 0 0 0 0 0 0 1 0 0 0 0 0 0 0 0 1 0 0 0 0 0 0 0 0 0 0 0 0 0
CO	0 0 0 0 0 0 0 0 0 0 0 0 0 1 0 0 0 0 0 0 0 0 0 0 1 0 0 0 1 0 0 0 0 1 0 0 0 0 0 0 0 1 0 0 0 0 0 1
CT	0 0 0 0 0 0 0 0 0 0 0 0 0 0 0 0 0 0 1 0 0 0 0 0 0 0 0 0 0 1 0 0 0 0 0 0 1 0 0 0 0 0 0 0 0 0 0 0
DE	0 0 0 0 0 0 0 0 0 0 0 0 0 0 0 0 0 0 0 0 0 0 0 0 0 0 0 1 0 0 0 0 0 0 0 1 0 0 0 0 0 0 0 0 0 0 0 0
FL	1 0 0 0 0 0 0 0 1 0 0 0 0 0 0 0 0 0 0 0 0 0 0 0 0 0 0 0 0 0 0 0 0 0 0 0 0 0 0 0 0 0 0 0 0 0 0 0
GA	1 0 0 0 0 0 0 1 0 0 0 0 0 0 0 0 0 0 0 0 0 0 0 0 0 0 0 0 0 0 1 0 0 0 0 0 0 1 0 1 0 0 0 0 0 0 0 0
ID	0 0 0 0 0 0 0 0 0 0 0 0 0 0 0 0 0 0 0 0 0 0 0 1 0 1 0 0 0 0 0 0 0 0 1 0 0 0 0 0 0 1 0 0 1 0 0 1
IL	0 0 0 0 0 0 0 0 0 0 0 1 1 0 1 0 0 0 0 0 0 0 1 0 0 0 0 0 0 0 0 0 0 0 0 0 0 0 0 0 0 0 0 0 0 0 1 0
IN	0 0 0 0 0 0 0 0 0 0 1 0 0 0 1 0 0 0 0 1 0 0 0 0 0 0 0 0 0 0 0 0 1 0 0 0 0 0 0 0 0 0 0 0 0 0 0 0
IA	0 0 0 0 0 0 0 0 0 0 1 0 0 0 0 0 0 0 0 0 1 0 1 0 1 0 0 0 0 0 0 0 0 0 0 0 0 0 1 0 0 0 0 0 0 0 1 0
KS	0 0 0 0 1 0 0 0 0 0 0 0 0 0 0 0 0 0 0 0 0 0 1 0 1 0 0 0 0 0 0 0 0 1 0 0 0 0 0 0 0 0 0 0 0 0 0 0
KY	0 0 0 0 0 0 0 0 0 0 1 1 0 0 0 0 0 0 0 0 0 0 0 0 0 0 0 0 0 0 0 0 1 0 0 0 0 0 0 0 0 0 0 1 0 1 0 0
LA	0 0 1 0 0 0 0 0 0 0 0 0 0 0 0 0 0 0 0 0 0 1 0 0 0 0 0 0 0 0 0 0 0 0 0 0 0 0 0 0 1 0 0 0 0 0 0 0
ME	0 0 0 0 0 0 0 0 0 0 0 0 0 0 0 0 0 0 0 0 0 0 0 0 0 0 1 0 0 0 0 0 0 0 0 0 0 0 0 0 0 0 0 0 0 0 0 0
MD	0 0 0 0 0 0 0 0 0 0 0 0 0 0 0 0 0 0 0 0 0 0 0 0 0 0 0 0 0 0 0 0 0 0 0 1 0 0 0 0 0 0 0 0 0 1 0 0
MA	0 0 0 0 0 1 0 0 0 0 0 0 0 0 0 0 0 0 0 0 0 0 0 0 0 0 1 0 0 1 0 0 0 0 0 0 0 0 0 0 0 0 1 0 0 0 0 0
MI	0 0 0 0 0 0 0 0 0 0 0 1 0 0 0 0 0 0 0 0 0 0 0 0 0 0 0 0 0 0 0 0 1 0 0 0 0 0 0 0 0 0 0 0 0 0 1 0
MN	0 0 0 0 0 0 0 0 0 0 0 0 1 0 0 0 0 0 0 0 0 0 0 0 0 0 0 0 0 0 0 1 0 0 0 0 0 0 1 0 0 0 0 0 0 0 1 0
MS	1 0 1 0 0 0 0 0 0 0 0 0 0 0 0 1 0 0 0 0 0 0 0 0 0 0 0 0 0 0 0 0 0 0 0 0 0 0 0 1 0 0 0 0 0 0 0 0
MO	0 0 1 0 0 0 0 0 0 0 1 0 1 1 0 0 0 0 0 0 0 0 0 0 1 0 0 0 0 0 0 0 0 1 0 0 0 0 0 0 0 0 0 0 0 0 0 0
MT	0 0 0 0 0 0 0 0 0 1 0 0 0 0 0 0 0 0 0 0 0 0 0 0 0 0 0 0 0 0 0 1 0 0 0 0 0 0 1 0 0 0 0 0 0 0 0 1
NE	0 0 0 0 1 0 0 0 0 0 0 0 1 1 0 0 0 0 0 0 0 0 1 0 0 0 0 0 0 0 0 0 0 0 0 0 0 0 1 0 0 0 0 0 0 0 0 1
NV	0 1 0 1 0 0 0 0 0 1 0 0 0 0 0 0 0 0 0 0 0 0 0 0 0 0 0 0 0 0 0 0 0 0 1 0 0 0 0 0 0 1 0 0 0 0 0 0
NH	0 0 0 0 0 0 0 0 0 0 0 0 0 0 0 0 1 0 1 0 0 0 0 0 0 0 0 0 0 0 0 0 0 0 0 0 0 0 0 0 0 0 1 0 0 0 0 0
NJ	0 0 0 0 0 0 1 0 0 0 0 0 0 0 0 0 0 0 0 0 0 0 0 0 0 0 0 0 0 1 0 0 0 0 0 1 0 0 0 0 0 0 0 0 0 0 0 0
NM	0 1 0 0 1 0 0 0 0 0 0 0 0 0 0 0 0 0 0 0 0 0 0 0 0 0 0 0 0 0 0 0 0 1 0 0 0 0 0 0 1 0 0 0 0 0 0 0
NY	0 0 0 0 0 1 0 0 0 0 0 0 0 0 0 0 0 0 1 0 0 0 0 0 0 0 0 1 0 0 0 0 0 0 0 1 0 0 0 0 0 0 1 0 0 0 0 0
NC	0 0 0 0 0 0 0 0 1 0 0 0 0 0 0 0 0 0 0 0 0 0 0 0 0 0 0 0 0 0 0 0 0 0 0 0 0 1 0 1 0 0 0 0 0 0 0 0
ND	0 0 0 0 0 0 0 0 0 0 0 0 0 0 0 0 0 0 0 0 1 0 0 1 0 0 0 0 0 0 0 0 0 0 0 0 0 0 1 0 0 0 0 0 0 0 0 0
OH	0 0 0 0 0 0 0 0 0 0 0 1 0 0 1 0 0 0 0 1 0 0 0 0 0 0 0 0 0 0 0 0 0 0 0 1 0 0 0 0 0 0 0 0 0 1 0 0
OK	0 0 1 0 1 0 0 0 0 0 0 0 0 1 0 0 0 0 0 0 0 0 1 0 0 0 0 0 1 0 0 0 0 0 0 0 0 0 0 0 1 0 0 0 0 0 0 0
OR	0 0 0 1 0 0 0 0 0 1 0 0 0 0 0 0 0 0 0 0 0 0 0 0 0 1 0 0 0 0 0 0 0 0 0 0 0 0 0 0 0 0 0 0 1 0 0 0
PA	0 0 0 0 0 0 1 0 0 0 0 0 0 0 0 0 0 1 0 0 0 0 0 0 0 0 0 1 0 1 0 0 1 0 0 0 0 0 0 0 0 0 0 0 0 1 0 0
RI	0 0 0 0 0 1 0 0 0 0 0 0 0 0 0 0 0 0 0 0 0 0 0 0 0 0 0 0 0 0 0 0 0 0 0 0 0 0 0 0 0 0 0 0 0 0 0 0
SC	0 0 0 0 0 0 0 0 1 0 0 0 0 0 0 0 0 0 0 0 0 0 0 0 0 0 0 0 0 0 1 0 0 0 0 0 0 0 0 0 0 0 0 0 0 0 0 0
SD	0 0 0 0 0 0 0 0 0 0 0 0 1 0 0 0 0 0 0 0 1 0 0 1 1 0 0 0 0 0 0 1 0 0 0 0 0 0 0 0 0 0 0 0 0 0 0 1
TN	1 0 1 0 0 0 0 0 1 0 0 0 0 0 0 0 0 0 0 0 0 1 0 0 0 0 0 0 0 0 1 0 0 0 0 0 0 0 0 0 0 0 0 1 0 0 0 0
TX	0 0 1 0 0 0 0 0 0 0 0 0 0 0 0 1 0 0 0 0 0 0 0 0 0 0 0 0 1 0 0 0 0 1 0 0 0 0 0 0 0 0 0 0 0 0 0 0
UT	0 1 0 0 1 0 0 0 0 1 0 0 0 0 0 0 0 0 0 0 0 0 0 0 0 1 0 0 0 0 0 0 0 0 0 0 0 0 0 0 0 0 0 0 0 0 0 1
VT	0 0 0 0 0 0 0 0 0 0 0 0 0 0 0 0 0 0 1 0 0 0 0 0 0 0 1 0 0 1 0 0 0 0 0 0 0 0 0 0 0 0 0 0 0 0 0 0
VA	0 0 0 0 0 0 0 0 0 0 0 0 0 0 1 0 0 0 0 0 0 0 0 0 0 0 0 0 0 0 0 0 0 0 0 0 0 0 0 1 0 0 0 0 0 1 0 0
WA	0 0 0 0 0 0 0 0 0 1 0 0 0 0 0 0 0 0 0 0 0 0 0 0 0 0 0 0 0 0 0 0 0 0 1 0 0 0 0 0 0 0 0 0 0 0 0 0
WV	0 0 0 0 0 0 0 0 0 0 0 0 0 0 1 0 0 1 0 0 0 0 0 0 0 0 0 0 0 0 0 0 1 0 0 1 0 0 0 0 0 0 0 1 0 0 0 0
WI	0 0 0 0 0 0 0 0 0 0 1 0 1 0 0 0 0 0 0 1 1 0 0 0 0 0 0 0 0 0 0 0 0 0 0 0 0 0 0 0 0 0 0 0 0 0 0 0
WY	0 0 0 0 1 0 0 0 0 1 0 0 0 0 0 0 0 0 0 0 0 0 0 1 1 0 0 0 0 0 0 0 0 0 0 0 0 0 1 0 0 1 0 0 0 0 0 0
