# Supplementary material for: A comparison of six analytical disease mapping techniques as applied to West Nile Virus in the coterminous United States
Source: Int J Health Geogr. 2005 Aug 2;4:18. doi: 10.1186/1476-072X-4-18 (PMC1215506; doi:10.1186/1476-072X-4-18)
Supplement: Additional File 2 — Data input and preparation for the SAR model estimation with SAS; SAR model estimation with SAS. SAS computer code, in which the input data file paths and file names may need to be changed (a), for estimating a simultaneous spatial autoregressive model (b). [file 1476-072X-4-18-S2.pdf]

2a: Data input and preparation for the SAR model estimation with SAS.

```
FILENAME INDATA 'C:\WNV-US-2003&2004.PRN';
FILENAME EIGEN  'C:\EIG-FROM-FIPS-ORDERING.PRN';
FILENAME CONN   'C:\US-STATES-BY-FIPS.CON';

DATA STEP1;
     INFILE INDATA; INPUT NAME$ C2003 D2003 C2004 D2004;
CASES =C2004; DEATHS=D2004;
IF CASES=0 THEN MCASES=1; ELSE MCASES=CASES;
IF CASES=0 THEN I0=1; ELSE I0=0;
IF NAME="DC" THEN DELETE;
* Y = LOG((DEATHS+0.14)/(CASES-DEATHS+0.30));
  Y = LOG((DEATHS+0.15)/(CASES-DEATHS+0.29));
IF CASES>0 THEN Y0=DEATHS/CASES; ELSE Y0=0;
RUN;
PROC SORT OUT=STEP1(REPLACE=YES); BY NAME; RUN;

DATA STEP1 (REPLACE=YES); SET STEP1;
     INFILE CONN;
     INPUT ID C1-C48;
     ARRAY CONY{48} CY1-CY48;
     ARRAY CONYO{48} CYO1-CYO48;
     ARRAY CONIO{48} CIO1-CIO48;
     ARRAY CON{48} C1-C48;
     CSUM = 0;
     DO I=1 TO 48;
          CSUM = CSUM + CON{I};
          CONY{I} = Y*CON{I};
          CONYO{I} = Y0*CON{I};
          CONIO{I}=I0*CON{I};
     END;
RUN;

PROC MEANS DATA=STEP1 NOPRINT; VAR CY1-CY48; OUTPUT OUT=CYOUT1 SUM=CY1-
CY48; RUN;
PROC TRANSPOSE DATA=CYOUT1 PREFIX=CY OUT=CYOUT2; VAR CY1-CY48; RUN;
PROC MEANS DATA=STEP1 NOPRINT; VAR CYO1-CYO48; OUTPUT OUT=CYOOUT1 SUM=CYO1-
CYO48; RUN;
PROC TRANSPOSE DATA=CYOOUT1 PREFIX=CYO OUT=CYOOUT2; VAR CYO1-CYO48; RUN;
PROC MEANS DATA=STEP1 NOPRINT; VAR CIO1-CIO48; OUTPUT OUT=CIOOUT1 SUM=CIO1-
CIO48; RUN;
PROC TRANSPOSE DATA=CIOOUT1 PREFIX=CI0 OUT=CIOOUT2; VAR CIO1-CIO48; RUN;
DATA EIGEN; INFILE EIGEN; INPUT IDE LAMBDAC LAMBDAW; LAMBDA=LAMBDAW; RUN;
PROC TRANSPOSE DATA=EIGEN PREFIX=TLAM OUT=CYOUT3; VAR LAMBDA; RUN;
DATA STEP1 (REPLACE=YES);
     IF _N_=1 THEN SET CYOUT3;
     SET STEP1;
     SET CYOUT2;
     SET CYOOUT2;
     SET CIOOUT2;
WY = CY1/CSUM;
WY0= CYO1/CSUM;
WI0= CIO1/CSUM;
RUN;
```

2b: SAR model estimation with SAS.

```
PROC NLIN DATA=STEP1 NOITPRINT METHOD=MARQUARDT MAXITER=500;
     PARMS RHO=0.5 B0=-3.0 B1=0;
     BOUNDS -1<RHO<1;

     ARRAY LAMBDAJ{48} TLAM1-TLAM48;
     JACOB = 0;
     DERJ = 0;
     DO I=1 TO 48;
          JACOB = JACOB + LOG(1 - RHO*LAMBDAJ{I});
          DERJ = DERJ + -LAMBDAJ{I}/(1 - RHO*LAMBDAJ{I});
     END;
     J=EXP(JACOB/48);
     DERJ = -DERJ/48;

     ZY = Y/J;

     MODEL ZY = (RHO*WY + B0*(1 - RHO) + B1*(I0 - RHO*WI0) )/J;
     OUTPUT OUT=TEMP2 PRED=YHAT R=YRESID;

     DER.RHO = ( (RHO*WY + B0*(1 - RHO) + B1*(I0 - RHO*WI0) - Y)*DERJ +
                                                        WY - B0 -
B1*WI0)/J;
RUN;
PROC UNIVARIATE DATA=TEMP2 NORMAL; VAR YRESID; RUN;
PROC REG; MODEL Y=YHAT; RUN;
```
