# Supplementary material for: A comparison of six analytical disease mapping techniques as applied to West Nile Virus in the coterminous United States
Source: Int J Health Geogr. 2005 Aug 2;4:18. doi: 10.1186/1476-072X-4-18 (PMC1215506; doi:10.1186/1476-072X-4-18)
Supplement: Additional File 3 — Minitab 14.13 code for computing spatial filter eigenvectors. Minitab computer code, in which the input data file paths and file names may need to be changed; the number of areal units is stored in K1, the geographic connectivity matrix is stored in M1, and the spatial filter eigenvectors are stored in M2. [file 1476-072X-4-18-S3.pdf]

3: Minitab 14.13 code for computing spatial filter eigenvectors.

```
READ c1-c48;
FILE 'C:\US-STATES-BY-FIPS.CON';
FORMAT(10X,48I2).
COPY C1-C48 M1
ERASE C1-C48
LET K1=48
SET C1
K1(1)
END
COPY C1 M2
TRANS M2 M3
MULT M2 M3 M2
LET K3=-1/K1
MULT K3 M2 M2
DIAG C1 M3
ADD M3 M2 M2
MULT M2 M1 M3
MULT M3 M2 M3
EIGEN M1 C1
EIGEN M3 C2 M2
PRINT C1 C2
END
```
