# Supplementary material for: A comparison of six analytical disease mapping techniques as applied to West Nile Virus in the coterminous United States
Source: Int J Health Geogr. 2005 Aug 2;4:18. doi: 10.1186/1476-072X-4-18 (PMC1215506; doi:10.1186/1476-072X-4-18)
Supplement: Additional File 4 — Data input, preparation, and estimation of the Gaussian spatial filter model with SAS. SAS computer code, in which the input data file paths and file names may need to be changed, for estimating a linear regression spatial filter model. [file 1476-072X-4-18-S4.pdf]

4: Data input, preparation, and estimation of the Gaussian spatial filter model with SAS.

```
FILENAME INDATA 'C:\WNV-US-2003&2004.PRN';
FILENAME EVECS  'C:\EVECS_ORDERED_BY_FIPS.TXT';
FILENAME MC     'C:\MC-BY-FIPS.TXT';
FILENAME CONN   'C:\US-STATES-BY-FIPS.CON';

DATA STEP1; INFILE INDATA; INPUT NAME$ C2003 D2003 C2004 D2004;
  CASES =C2003; DEATHS=D2003;
  IF CASES=0 THEN I0=1; ELSE I0=0;
  IF NAME="DC" THEN DELETE;
  Y = LOG((DEATHS+0.14)/(CASES-DEATHS+0.30));
* Y = LOG((DEATHS+0.15)/(CASES-DEATHS+0.29));
RUN;
PROC SORT OUT=STEP1(REPLACE=YES); BY NAME; RUN;
DATA STEP2; INFILE EVECS; INPUT IDE E1-E48; RUN;
DATA STEP2(REPLACE=YES); SET STEP2; SET STEP1; RUN;

PROC REG DATA=STEP2 OUTEST=COEF; MODEL Y = I0 E1-E31
              /SELECTION=STEPWISE INCLUDE=1 SLE=0.10 SLS=0.10;
             OUTPUT OUT=TEMP P=YHAT R=YRESID; RUN;
PROC TRANSPOSE DATA=COEF PREFIX=B OUT=COEF2; VAR E1-E31; RUN;
DATA COEF(REPLACE=YES); INFILE MC; INPUT ID LAM_MCM MC; RUN;
PROC MEANS NOPRINT; VAR MC; OUTPUT OUT=OUTMCMAX MAX=MCMAX; RUN;
DATA COEF(REPLACE=YES); SET COEF; IF _N_=1 THEN SET OUTMCMAX;
     IF MC/MCMAX<0.25 THEN DELETE; RUN;
DATA COEF(REPLACE=YES); SET COEF; SET COEF2;
IF B1='.' THEN DELETE;
BSQ=B1**2; EMC=BSQ*MC; P=1;
RUN;
PROC MEANS SUM NOPRINT; VAR LAM_MCM P EMC BSQ; OUTPUT OUT=EMC SUM=SUML P
SPANUM SPADEN; RUN;
PROC STANDARD DATA=TEMP MEAN=0 STD=1 OUT=TEMP(REPLACE=YES); VAR YRESID;
RUN;

DATA STEP2(REPLACE=YES); SET STEP2; INFILE CONN; INPUT ID C1-C48; RUN;
DATA STEP2(REPLACE=YES); SET STEP2; SET TEMP(KEEP=YRESID);
     ARRAY CONN{48} C1-C48;
     ARRAY ZC{48} ZC1-ZC48;
     CSUM=0;
       DO I=1 TO 48;
          CSUM = CSUM + CONN{I};
          ZC{I} = YRESID*CONN{I};
       END;
Z=YRESID; X0=1;
RUN;
PROC MEANS SUM NOPRINT; VAR CSUM X0; OUTPUT OUT=CSUM SUM=CSUM N; RUN;
PROC MEANS DATA=STEP2 NOPRINT; VAR ZC1-ZC48; OUTPUT OUT=ZCOUT1 SUM=ZC1-
ZC48; RUN;
PROC TRANSPOSE DATA=ZCOUT1 PREFIX=ZC OUT=ZCOUT2; VAR ZC1-ZC48; RUN;
DATA STEP2(REPLACE=YES); SET STEP2; SET ZCOUT2(KEEP=ZC1);
ZC=ZC1; DROP ZC1; RUN;
PROC REG DATA=STEP2 OUTEST=DEN NOPRINT; MODEL CSUM=X0/NOINT; RUN;
PROC REG DATA=STEP2 OUTEST=NUM NOPRINT; MODEL ZC=Z/NOINT; RUN;
DATA STEP3; SET DEN; SET NUM; SET CSUM(KEEP=CSUM N);
                              SET EMC(KEEP=SUML SPANUM SPADEN P);
MC=Z/X0; EMC = -(1+(N/CSUM)*SUML)/(N-P-1);
ZMC=(MC-EMC)/SQRT(2/CSUM);
MCSPA=SPANUM/SPADEN;
RUN;
PROC PRINT; VAR MC EMC ZMC MCSPA; RUN;
```
