# Supplementary material for: A comparison of six analytical disease mapping techniques as applied to West Nile Virus in the coterminous United States
Source: Int J Health Geogr. 2005 Aug 2;4:18. doi: 10.1186/1476-072X-4-18 (PMC1215506; doi:10.1186/1476-072X-4-18)
Supplement: Additional File 5 — Data input, preparation, and estimation of the logistic spatial filter model with SAS. SAS computer code, in which the input data file paths and file names may need to be changed, for estimating a generalized linear (logistic) regression spatial filter model. [file 1476-072X-4-18-S5.pdf]

5: Data input, preparation, and estimation of the logistic spatial filter model with SAS.

```
FILENAME INDATA 'C:\WNV-US-2003&2004.PRN';
FILENAME EVECS  'C:\EVECS_ORDERED_BY_FIPS.TXT';

DATA STEP1;
     INFILE INDATA;
     INPUT NAME$ C2003 D2003 C2004 D2004;
CASES =C2004; DEATHS=D2004;
IF CASES=0 THEN I0=1; ELSE I0=0;
IF NAME="DC" THEN DELETE;
RUN;
PROC SORT OUT=STEP1(REPLACE=YES); BY NAME; RUN;

DATA STEP2; INFILE EVECS LRECL=1024; INPUT IDE E1-E48; RUN;
DATA STEP2(REPLACE=YES); SET STEP2; SET STEP1; RUN;

DATA STEP3; SET STEP1; SET STEP2;
IF CASES>0 THEN Y=DEATHS/CASES; ELSE Y=0;
IF CASES=0 THEN CASES=1;
RUN;

PROC LOGISTIC; MODEL DEATHS/CASES=I0 E1-E31/SELECTION=STEPWISE INCLUDE=1
SLSTAY=0.1; RUN;

/*
PROC LOGISTIC; MODEL DEATHS/CASES=I0 E1 E4 E5 E6 E8 E10 E16 E20 E24 E29 E33
E35/SELECTION=STEPWISE INCLUDE=1 SLSTAY=0.1; RUN;
PROC GENMOD; MODEL DEATHS/CASES=I0 E1 E6 E11 E15 E18 E25/DIST=BIN; OUTPUT
OUT=TEMP3 P=YHAT2 XBETA=XBETA; RUN;
*/

PROC GENMOD; MODEL DEATHS/CASES=I0 E1 E3 E15/DIST=BIN; OUTPUT OUT=TEMP3
P=YHAT2 XBETA=XBETA; RUN;

PROC REG; MODEL Y=YHAT2; RUN;
DATA TEMP3(REPLACE=YES); SET TEMP3;
IF CASES>0 THEN YRESID2=DEATHS/CASES-YHAT2; ELSE YRESID2=0;
RUN;
PROC UNIVARIATE NORMAL; VAR YRESID2; RUN;
```
