# Supplementary material for: A comparison of six analytical disease mapping techniques as applied to West Nile Virus in the coterminous United States
Source: Int J Health Geogr. 2005 Aug 2;4:18. doi: 10.1186/1476-072X-4-18 (PMC1215506; doi:10.1186/1476-072X-4-18)
Supplement: Additional File 6 — Data input, preparation, and pseudo-likelihood estimation of the auto-logistic model with SAS. SAS computer code, in which the input data file paths and file names may need to be changed, for estimating a generalized linear auto-logistic regression model. [file 1476-072X-4-18-S6.pdf]

6: Data input, preparation, and pseudo-likelihood estimation of the autologistic model with SAS.

```
FILENAME INDATA 'C:\WNV-US-2003&2004.PRN';
FILENAME CONN   'C:\US-STATES-BY-FIPS.CON';

DATA STEP1;
     INFILE INDATA;
       INPUT NAME$ C2003 D2003 C2004 D2004;
CASES =C2003;
IF CASES=0 THEN MCASES=1; ELSE MCASES=CASES;
DEATHS=D2003;
IF CASES>0 THEN Y0=DEATHS/CASES; ELSE Y0=0;
IF CASES=0 THEN I0=1; ELSE I0=0;
IF NAME="DC" THEN DELETE;
RUN;
PROC SORT OUT=STEP1(REPLACE=YES); BY NAME; RUN;

DATA STEP1 (REPLACE=YES);
     SET STEP1;
     INFILE CONN;
     INPUT ID C1-C48;
     ARRAY CONYO{48} CYO1-CYO48;
     ARRAY CON{48} C1-C48;
     CSUM = 0;
     DO I=1 TO 48;
          CSUM = CSUM + CON{I};
          CONYO{I} = Y0*CON{I};
     END;
RUN;
PROC MEANS DATA=STEP1 NOPRINT;
     VAR CYO1-CYO48;
     OUTPUT OUT=CYOOUT1 SUM=CYO1-CYO48;
RUN;
PROC TRANSPOSE DATA=CYOOUT1 PREFIX=CYO OUT=CYOOUT2;
     VAR CYO1-CYO48;
RUN;
DATA STEP1 (REPLACE=YES);
     SET STEP1;
     SET CYOOUT2;
WY0= CYO1/CSUM;
RUN;

PROC GENMOD DATA=STEP1; MODEL DEATHS/MCASES=I0 WY0; OUTPUT OUT=TEMP P=YHAT;
RUN;
DATA TEMP(REPLACE=YES); SET TEMP;
YRESID=Y0-YHAT;
RUN;
PROC UNIVARIATE NORMAL; VAR YRESID; RUN;
PROC REG; MODEL Y0=YHAT; RUN;

DATA STEP1(REPLACE=YES); SET STEP1;
YEST = 1 - 1/(1 + EXP(-0.92976 + 1.85924*WY0 - 17.58*I0));
YERESID=Y0-YEST;
RUN;
PROC UNIVARIATE NORMAL; VAR YERESID; RUN;
PROC REG; MODEL Y0=YEST; RUN;
```
