# Supplementary material for: A comparison of six analytical disease mapping techniques as applied to West Nile Virus in the coterminous United States
Source: Int J Health Geogr. 2005 Aug 2;4:18. doi: 10.1186/1476-072X-4-18 (PMC1215506; doi:10.1186/1476-072X-4-18)
Supplement: Additional File 7 — 2003 data input and estimation of the PCAR logistic spatial filter model with GeoBUGS. [file 1476-072X-4-18-S7.pdf]

7: 2003 data input and estimation of the PCAR logistic spatial filter model with GeoBUGS.

```
#US WNV
#n=48

# Model
model {
      # Set up 'data' to define spatial dependence structure
      for(i in 1 : N) {
            m[i] <- 1
                # /num[i] would be scaling factor for variance in each cell
      }

cumsum[1] <- 0
      for(i in 2:(N+1)) {
            cumsum[i] <- sum(num[1:(i-1)])
      }
      for(k in 1 : sumNumNeigh) {
            for(i in 1:N) {
                 pick[k,i] <- step(k-cumsum[i]-epsilon)*step(cumsum[i+1]-k)
         # pick[k,i]=1 if cumsum[i]<k<=cumsum[i=1]; otherwise, pick[k,i]=0
            }
            C[k] <- 1/inprod(m[], pick[k,])
         # weight for each pair of neighbours; /inprod(num[], pick[k,])
      }
      epsilon <- 0.0001

# Likelihood
      for (i in 1 : N) {

          D[i] ~ dbin(p[i],WNV[i])
          p[i] <- exp(beta1*I0[i] + S[i])/(1+ exp(beta1*I0[i] + S[i]))
             theta[i] <- alpha
      }

# Proper CAR prior distribution for spatial random effects:
      S[1:N] ~ car.proper(theta[], C[], adj[], num[], m[], prec, gamma)
# Other priors:
      alpha ~ dnorm(0, 0.0001)
      beta1 ~ dnorm(0,0.0001)
      prec  ~ dgamma(0.5, 0.0005)                     # prior on precision
      std <- sqrt(1/prec)                             # variance
      gamma.min <- min.bound(C[], adj[], num[], m[])
      gamma.max <- max.bound(C[], adj[], num[], m[])
      gamma ~ dunif(gamma.min, gamma.max)
}

list(N=48,
WNV = c(37, 13, 25, 3, 2947, 17, 17, 94, 50, 1, 54, 47,
 147, 91, 14, 124, 1, 73, 17, 19, 148, 87, 64, 222,
 1942, 2, 3, 34, 209, 71, 24, 617, 108, 79, 1, 237,
 7, 6, 1039, 26, 720, 1, 3, 26,10, 2, 17, 375
),

D = c(3, 1, 0, 0, 63, 0, 2, 6, 4, 0, 1, 4,
 6, 4, 1, 8, 0, 8, 1, 2, 4, 1, 8, 4,
 29, 0, 0, 3, 4, 11, 2, 5, 8, 0, 0, 8,
 1, 0, 14, 1, 37, 0, 0, 1, 0, 0, 0, 9
),
```

```
I0 = c(0, 0, 0, 0, 0, 0, 0, 0, 0, 0, 0, 0, 0, 0, 0, 0,
 1, 0, 0, 0, 0, 0, 0, 0, 0, 0, 0, 0, 0, 0, 0, 0, 0, 0,
 1, 0, 0, 0, 0, 0, 0, 0, 0, 0, 1, 0, 0, 0),
num = c(4, 6, 6, 3, 7, 3, 3, 2, 5, 6,
5, 4, 6, 4, 7, 3, 6, 4, 5, 3,
4, 4, 8, 4, 6, 5, 3, 3, 4, 5,
4, 3, 5, 4, 4, 6, 2, 2, 4, 8,
4, 6, 3, 5, 2, 5, 1, 6),
adj = c(40, 22, 9, 8,
41, 34, 23, 14, 5, 3,
41, 40, 23, 22, 16, 2,
39, 29, 26,
48, 42, 34, 29, 25, 14, 2,
37, 30, 19,
36, 28, 18,
9, 1,
40, 38, 31, 8, 1,
48, 45, 42, 39, 26, 24,
35, 23, 15, 13, 12,
33, 20, 15, 11,
35, 25, 23, 21, 17, 11,
25, 23, 5, 2,
46, 44, 40, 33, 23, 12, 11,
41, 22, 3,
48, 32, 25, 24, 21, 13,
46, 44, 36, 7,
43, 37, 30, 27, 6,
35, 33, 12,
35, 32, 17, 13,
40, 16, 3, 1,
40, 25, 15, 14, 13, 11, 3, 2,
48, 32, 17, 10,
48, 23, 17, 14, 13, 5,
42, 39, 29, 10, 4,
47, 43, 19,
36, 30, 7,
42, 34, 26, 4,
43, 36, 28, 19, 6,
44, 40, 38, 9,
24, 21, 17,
46, 36, 20, 15, 12,
41, 29, 5, 2,
21, 20, 13, 11,
46, 33, 30, 28, 18, 7,
19, 6,
31, 9,
45, 26, 10, 4,
44, 31, 23, 22, 15, 9, 3, 1,
34, 16, 3, 2,
48, 34, 29, 26, 10, 5,
30, 27, 19,
46, 40, 31, 18, 15,
39, 10,
44, 36, 33, 18, 15,
27,
42, 25, 24, 17, 10, 5),   sumNumNeigh = 212)

# Initial values
list( alpha=-3, beta1=0, prec=1,
S=c(0,0,0,0,0,0,0,0,0,0,0,0,0,0,0,0,0,0,0,0,0,0,0,0,
    0,0,0,0,0,0,0,0,0,0,0,0,0,0,0,0,0,0,0,0,0,0,0,0) , gamma=-0.1)
```
