# Supplementary material for: A comparison of six analytical disease mapping techniques as applied to West Nile Virus in the coterminous United States
Source: Int J Health Geogr. 2005 Aug 2;4:18. doi: 10.1186/1476-072X-4-18 (PMC1215506; doi:10.1186/1476-072X-4-18)
Supplement: Additional File 8 — 2003 data input and estimation of the logistic spatial filter model with WinBUGS. [file 1476-072X-4-18-S8.pdf]

8: 2003 data input and estimation of the logistic spatial filter model with WinBUGS.

```
#US WNV
#n=48

# Model
model {
       # Likelihood
       for (i in 1 : N) {

           D[i] ~ dbin(p[i],C[i])
           p[i] <- exp(alpha + beta1*I0[i] + beta2*SF[i] + S[i])/(1+ exp(alpha +
beta1*I0[i] + beta2*SF[i] + S[i]))

# Exchangeable prior on unstructured random effects
             S[i] ~ dnorm(0, tau.s)
       }

       # Other priors:
       alpha ~ dnorm(0, 0.0001)
       beta1 ~ dnorm(0,0.0001)
       beta2 ~ dnorm(0,0.0001)
       tau.s  ~ dgamma(0.5, 0.0005)        # prior on precision
       vs <- 1/tau.s                                    # variance
       sigma <- sqrt(1 / tau.s)                      # standard deviation
}

# Data
list(N = 48,

D = c(
 3, 1, 0, 0, 63, 0, 2, 6, 4, 0, 1, 4, 6, 4, 1, 8,
 0, 8, 1, 2, 4, 1, 8, 4, 29, 0, 0, 3, 4, 11, 2, 5,
 8, 0, 0, 8, 1, 0, 14, 1, 37, 0, 0, 1, 0, 0, 0, 9
),

C = c(
 37, 13, 25, 3, 2947, 17, 17, 94, 50, 1, 54, 47, 147, 91, 14, 124,
 1, 73, 17, 19, 148, 87, 64, 222, 1942, 2, 3, 34, 209, 71, 24, 617,
 108, 79, 1, 237, 7, 6, 1039, 26, 720, 1, 3, 26, 1, 2, 17, 375
),

I0 = c(
 0, 0, 0, 0, 0, 0, 0, 0, 0, 0, 0, 0, 0, 0, 0, 0,
 1, 0, 0, 0, 0, 0, 0, 0, 0, 0, 0, 0, 0, 0, 0, 0,
 0, 0, 1, 0, 0, 0, 0, 0, 0, 0, 0, 0, 1, 0, 0, 0
),

SF = c(
 0.7255556684, -1.364778005, 0.0877110147, 0.358846536, -0.704452031, 0.1015734395,
0.6716738365, 0.1806074953,
 -0.225413251, 0.2605519194, -0.380516602, 0.492411853, -0.774055149, -0.270611963,
0.9499003118, 0.0369722026,
 0.0126955038, 0.5731985886, 0.3397002009, 0.1271316419, -0.106219616,
0.3113324856, 1.4524227708, -0.820385999,
 -1.155664451, -0.66884873, 0.358528261, 0.4107806143, -0.306890388, 1.234000249,
0.3111141977, -0.79016798,
 0.4958229527, -2.144903123, -0.137140318, 0.2004512091, 0.1622827723,
0.1328762633, -1.079974427, 0.9049706433,
 -0.058392246, -0.845419619, 0.1353588677, 0.2502594797, 0.0037591156,
1.2050886368, -0.356807062, -0.697828434
)
)

# Initial values
list( alpha=0, beta1=0, beta2=1, tau.s=1,
S=c(0,0,0,0,0,0,0,0,0,0,0,0,0,0,0,0,0,0,0,0,0,0,0,0,
    0,0,0,0,0,0,0,0,0,0,0,0,0,0,0,0,0,0,0,0,0,0,0,0) )
```
